# Symptoms of a broken system: the gender gaps in COVID-19 decision-making

**DOI:** 10.1136/bmjgh-2020-003549

**Published:** 2020-10-01

**Authors:** Kim Robin van Daalen, Csongor Bajnoczki, Maisoon Chowdhury, Sara Dada, Parnian Khorsand, Anna Socha, Arush Lal, Laura Jung, Lujain Alqodmani, Irene Torres, Samiratou Ouedraogo, Amina Jama Mahmud, Roopa Dhatt, Alexandra Phelan, Dheepa Rajan

**Affiliations:** 1 Cardiovascular Epidemiology Unit, Cambridge University, Cambridge, UK; 2 Women in Global Health, Washington, District of Columbia, USA; 3 UHC2030 Alliance, Health Systems Governance Collaborative, World Health Organization, Geneva, Switzerland; 4 Vayu Global Health Foundation, Boston, Massachusetts, USA; 5 Leipzig University, Leipzig, Germany; 6 Education and Agriculture Together (EAT) Foundation, Oslo, Norway; 7 Fundacion Octaedro, Quito, Ecuador; 8 McGill University, Montreal, Quebec, Canada; 9 Institut National de Santé Publique du Québec (INSPQ), Montreal, Quebec, Canada; 10 Women in Global Health, Garowe, Somalia; 11 Department of Women’s and Children’s Health, Uppsala University Department of International Maternal and Child Health, Uppsala, Sweden; 12 Center for Global Health Science & Security, Georgetown University, Washington, District of Columbia, USA

**Keywords:** health policy

**Summary box**

- Despite numerous global and national commitments to gender-inclusive global health governance, COVID-19 followed the usual modus operandi –excluding women’s voices. A mere 3.5% of 115 identified COVID-19 decision-making and expert task forces have gender parity in their membership while 85.2% are majority men.
- With 87 countries included in this analysis, information regarding task force composition and membership criteria was not easily publicly accessible for the majority of United Nations Member States, impeding the ability to hold countries accountable to previously made commitments.
- Lack of representation is one symptom of a broken system where governance is not inclusive of gender, geography, sexual orientation, race, socio-economic status or disciplines within and beyond health – ultimately excluding those who offer unique perspectives and expertise.
- Functional health systems require radical and systemic change that ensures gender-responsive and intersectional practices are the norm – rather than the exception.
- Open, inclusive and transparent communication and decision-making must be prioritised over closed-door or traditional forms of governance.
- Data collection and governance policies must include sex and gender data, and strive for an intersectionality approach that includes going beyond binary representation in order to produce results that are inclusive of the full gender spectrum.

A growing chorus of voices are questioning the glaring lack of women in COVID-19 decision-making bodies. Men dominating leadership positions in global health has long been the default mode of governing. This is a symptom of a broken system where governance is not inclusive of any type of diversity, be it gender, geography, sexual orientation, race, socio-economic status or disciplines within and beyond health – excluding those who offer unique perspectives, expertise and lived realities. This not only reinforces inequitable power structures but undermines an effective COVID-19 response – ultimately costing lives.

By providing quantitative data, we critically assess the gender gap in task forces organised to prevent, monitor and mitigate COVID-19, and emphasise the paramount exclusion of gender-diverse voices.

## Retreating to the non-inclusive default mode of governance

The global community was unprepared as COVID-19 struck. As a result, countries swiftly established expert and decision-making structures through traditional processes: reaching out to government ministry directors, prominent experts and heads of well-known institutions. Most of these positions are typically held by men, as evidenced by our analysis of 115 expert and decision-making COVID-19 task forces from 87 countries: 85.2% of identified national task forces (n=115) contain mostly men, only 11.4% contain predominantly women and a mere 3.5% exhibit gender parity.* Similarly, 81.2% (n=65) of these task forces were headed by men (table 1).

**Table 1:** Identified national COVID-19 task forces

#	Country (Reference)	Name of the task force convened	Type of task force	Gender	Women head of force	Public	Women head of gov	Note
1	Albania^23^	**Technical Committee of Experts(for Covid-19**) “Komiteti i Ekspertëve”	Expert	8W; 3M (11 total) 72.7%W	Unknown	Yes	No, Edi Rama	N/A
2	Algeria^24^	**National Committee for Monitoring and Follow-up of the Corona Virus (Covid-19**) اللجنة الوطنية العلمية لرصد ومتابعة تفشي فيروس كورون	Expert	0W; 11M (11 total) 0%W	No, Abderahmane Ben Bouzid	Yes	No, Abdelaziz Djerad	N/A
3	Argentina^25 26^	**Committee of medical and scientific experts** “Comité de expertos médicos y cientificos”	Expert	4W; 6M (10 total) 40%W	Unknown	Yes	No, Alberto Fernández	N/A
4	Armenia^27 28^	**Interdepartmental Commission for Coordinating the Prevention of the Spread of the new Coronavirus** “միջգերատեսչական հանձնաժողով”	Decision-making	4W; 10M (14 total) 28.6%W	No, Arsen Torosyan	Yes	No, Nikol Pashinyan	N/A
		**IT working group modelling spread of coronavirus in Armenia** (No formal name)	Expert	0W; 12M (12 total) 0%W	Unknown	Yes		Photo reference(s) were used to determine gender composition.
5	Australia^29–32^	**Australian National COVID-19 Coordination Commission**	Decision-making	2W; 6M (8 total) 25%W	No, Neville Power	Yes	No, Scott Morrison	N/A
		**Australian Health Protection Principal Committee**	Decision-making	3W; 6M (9 total) 33.3%W	No, Brendan Murphy	Yes		N/A
6	Austria^33^	**Coronavirus Taskforce** “Hausinternen Stabs der Coronavirus-Taskforce”	Decision-making	6W; 4M (10 total) 60%W	Unknown	Yes	No, Sebastian Kurz	N/A
		**Advisory Team to the Coronavirus Taskforce** “Beraterstabs der Coronavirus-Taskforce”	Expert	5W; 13M (18 total) 27.8%W	Unknown	Yes		N/A
7	Bahamas^34 35^	**National Coordination Committee on COVID-19**	Decision-making	6W; 11M (17 total) 35.3%	Yes (co-chair), Pearl McMillan and Matt Aubry	Yes	No, Hubert Minnis	N/A
8	Bahrain^36^	**National Taskforce for Combating Coronavirus (COVID-19**) الفريق الوطني للتصدي لفيروس كورونا	Decision-making and expert	2W; 3M (5 total) 40%	Unknown	Yes	No, Khalifa bin Salman Al Khalifa	N/A
9	Bangladesh^37^	**National Committee for Prevention and Control of Covid-19** “জাতীয় কমিটি কোভিড -১৯ এর প্রতিরোধ ও নিয়ন্ত্রণের জন্য”	Decision-making	4W; 28M (32 total) 12.5%W	No, Zahid Maleque	Yes	Yes, Sheikh Hasina	N/A
10	Belgium^38 39^	**Scientific Committee for Coronavirus** “Wetenschappelijk comité Coronavirus” “Comité scientifique Coronavirus”	Expert	3W; 2M (5 total) 60%W	No, Steven van Gucht	Yes	Yes, Sophie Wilmes	N/A
11	Benin^40^	**Interdepartmental Committee** “Comité interministériel”	Decision-making	0W; 4M (4 total) 0%W	No, unknown	Yes	No, Patrice Talon	N/A
12	Bhutan^41^	**Health Emergency Management Committee**	Decision-making	2W; 11M (13 total) 15.4% W	Yes, Lyonpo Dechen Wangmo	Yes	No, Lotay Tshering	N/A
		**Technical Advisory Group**	Expert	2W; 11M (13 total) 15.4%W	No, Sithar Dorjee	Yes		N/A
13	Bolivia^42^	**Scientific Advisory Council** “Consejo Científico Asesor para la lucha contra COVID-19 en Bolivia”	Expert	2W; 6M (8 total) 25%W	No, Carlos Javier Cuellar	Yes	Yes, Jeanine Añez	N/A
14	Botswana^43^	**COVID-19 Task Force Team**	Expert	0W; 4M (4 total) 0%W	No, unknown	Yes	No, Mokgweetsi Masisi	N/A
15	Brazil^44–49^	**Interministerial Executive Group on Public Health Emergency of National and International Importance** “Grupo Executivo Interministerial de Emergência em Saúde Pública de Importância Nacional e Internacional”	Decision-making	1W; 8M (9 total) 11.1%W	Unknown	Yes	No, Jair Bolsonaro	N/A
		**Crisis Committee for Supervision and Monitoring of Covid-19 Impacts** “Comitê de Crise para Supervisão e Monitoramento dos Impactos da Covid-19”	Unclear	1W; 21M (22 total) 4.5%W	Unknown	Yes		N/A
16	Bulgaria^50^	**Medical Council** “медицинския мозъчен тръст"	Expert	5W; 11M (16 total) 31.3%W	Unknown	Yes	No, Boyko Borisov	Committee was dispersed (functioned until 4 April)
17	Burkina Faso^51^	**Name unknown**	Decision-making & Expert	5W; 14M (19 total) 26.3%W	Unknown	No	No, Christophe Joseph Marie Dabiré	N/A
18	Cape Verde^52–54^	**Council of Ministers** “Conselho de Ministros”	Decision-making	3W; 12M (15 total) 20%W	Unknown	Yes	No, Ulisses Correia e Silva	N/A
19	Canada^55–59^	**Cabinet Committee on the federal response to the coronavirus disease (COVID-19**)	Decision-making	4W; 4M (8 total) 50%W	Yes, Chrystia Freeland	Yes	No, Justin Trudeau	N/A
		**Special Advisory Committee on COVID-19**	Expert	12W; 11M (23 total) 52.2%W	Yes, Theresa Tam and Sadiq Shahab	Yes		N/A
20	Chad^60^	**Scientific Committee for Covid-19** “Comité Scientifique Covid-19”	Expert	4W; 33M (37 total) 10.8%W	Unknown	No	No, Idriss Déby	N/A
21	Chile^61^	**Scientific Advisory Council for Covid-19** “Consejo científico asesor por Covid-19”	Expert	4W; 6M (10 total) 40%W	Unknown	Yes	No, Sebastián Piñera	N/A
22	China 62–66	**Central Leading Group on Responding to the Novel Coronavirus Disease Outbreak** “Xīnxíng guānzhuàng bìngdú gǎnrǎn xìng fèiyán zhōngyāng lǐngdǎo xiǎozǔ”	Decision-making	1W; 8M (9 total) 11.1%W	No, Li Keqiang	Yes	No, Li Keqiang	N/A
		**Central Steering Group** (unofficial name) “Zhōngyāng zhǐdǎo xiǎozǔ”	Other	2W; 10M (12 total) 16.7%W	Yes, Sun Chunlan	Yes		N/A
23	Colombia^67^	**Contingency plan to respond to the emergency by COVID-19** “Plan de contingencia para responder ante la emergencia por COVID-19”	Decision-making	5W; 9M (14 total) 35.7%W	Unknown	Yes	No, Iván Duque	N/A
24	Comoros^68^	**Comité National de Coordination – Cadre de Gestion et de Coordination de la Crise du Covid-19** “National Coordination Committee - Management and Coordination Framework for the Covid-19 Crisis”	Decision-making & expert	2W; 33M (35 total) 5.7%W	Unknown	No	No, Azali Assoumani	N/A
25	Congo^69^	**National coordination for the management of the coronavirus pandemic** “Coordination nationale de gestion de la pandémie de coronavirus (COVID-19)”	Decision-making	3W; 12M (15 total) 20%W	Yes, Jacqueline Lydia Mikolo	Yes	No, Clément Mouamba	N/A
26	Costa Rica^70^	**The National Commission for Risk Prevention and Emergency Attention** “La Comisión Nacional de Prevención de Riesgos y Atención de Emergencias (CNE)”	Decision-making	3W; 17M (20 total) 15%W	No, Alexander Solís Delgado	Yes	No, Carlos Alvarado Quesada	N/A
27	Côte d'Ivoire^71^	**The scientific committee** “Le comité scientifique”	Expert	1W; 5M (6 total) 16.7%W	Unknown	No	No, Amadou Gon Coulibaly	N/A
28	Cuba^72 73^	**The working group for the prevention and control of COVID-19** “El grupo de trabajo para la prevención y el control de la COVID-19”	Decision-making	5W; 10M (15 total) 33.3%W	No, Miguel Díaz-Canel Bermúdez, Manuel Marrero Cruz and Salvador Valdés Mesa	Yes	No, Manuel Marrero Cruz	Photo reference(s) were used to determine gender composition. This may not be complete.
29	Cyprus^74–76^	**Council of Ministers**	Decision-making	1W; 11M (12 total) 8.3%W	No, Nicos Anastasiades	Yes	No, Nicos Anastasiades	N/A
30	Democratic People’s Republic of Korea^77 78^	(**enlarged) Political Bureau**	Decision-making	1W; 47M (48 total) 2.1%W	No, Kim Jong-un	Yes	No, Kim Jong-un	Photo reference(s) were used to determine gender composition.
31	Democratic Republic of the Congo^79–81^	**Multisectoral crisis committee** “Comité multisectoriel de crise”	Decision-making	3W; 16M (19 total) 15.8%W	No, Sylvestre Ilunga Ilunkamba	Yes	No, Sylvestre Ilunga Ilunkamba	Photo reference(s) were used to determine gender composition.
		**Management Committee of the National Solidarity Fund against Coronavirus** “Comité de gestion du Fonds national de solidarité contre le Coronavirus (FNSCC)”	Other	2W; 10M (12 total) 16.7%W	No, Révérend Dominique Mukanya	Yes		N/A
32	Djibouti^82 83^	**Steering committee** “Comité de pilotage”	Decision-making	1W; 9M (10 total) 10%W	No, Abdoulkader Kamil Mohamed	Yes	No, Abdoulkader Kamil Mohamed	N/A
33	Dominican Republic^84^	**Emergency and Health Management Committee to Combat COVID-19** “Comité de Emergencia y Gestión Sanitaria para el Combate del COVID-19”	Decision-making and expert	1W; 6M (7 total) 14.3%W	No, Amado Alejandro Baez	Yes	No, Danilo Medina	N/A
34	Ecuador^85 86^	**COVID-19 Technical Team** “Mesa Técnica COVID-19”	Expert	8W; 23M (31 total) 25.8%W	Unknown	Yes	No, Lenín Moreno	N/A
		**National Epidemiological Coordination** “Coordinación Nacional de Vigilancia Epidemiológica”	Expert	3W; 2M (5 total) 60%W	Unknown	Yes		N/A
35	Estonia^87^	**Government Commission** “Valitsuskomisjon”	Decision-making	1W; 9M (10 total) 10%W	No, Jüri Ratas	Yes	No, Jüri Ratas	N/A
		**Scientific Advisory Board** “Teadusnõukoda”	Expert	3W; 2M (5 total) 60%W	Yes, Irja Lutsar	Yes		N/A
36	Eswatini^88^	**National Emergency Management Committee**	Decision-making	3W; 8M (11 total) 27.27%W	No, Themba N. Masuku	Yes	No, Ambrose Mandvulo Dlamini	N/A
		**National Emergency Task Force**	Other	7W; 21M (28 total) 25%W	Unknown	Yes		N/A
37	Ethiopia^89 90^	**COVID19 National Ministerial Committee**	Decision-making	2W; 2M (four total) 50%W	Unknown	Yes	No, Abiy Ahmed	N/A
		**National COVID-19 advisory committee**	Expert	6W; 17M (23 total) 26.1%M	Unknown	Yes		N/A
38	Finland^91 92^	**Working group on essential work-related travel and other traffic**	Other	11W; 7M (18 total) 61.1%W	Yes, Sonja Hämäläinen	Yes	Yes, Sanna Marin	N/A
		**Working group to examine realisation of children’s rights in aftermath of coronavirus**	Other	4W; 2M (6 total) 66.6%W	No, Esa Iivonen	Yes		N/A
39	France^93–96^	**The Covid-19 Scientific Council** “Le Conseil Scientifique Covid-19”	Expert	2W; 9M (11 total) 18.2%W	No, Jean-François Delfraissy	Yes	No, Édouard Philippe	N/A
		**Research and expertise analysis committee** “Comité analyse recherche et expertise”	Expert	5W; 7M (12 total) 41.7%W	Yes, Françoise Barré-Sinoussi	Yes		N/A
40	Gabon^97^	**Scientific committee on the Coronavirus epidemic** “Comité scientifique sur l’épidémie à Coronavirus (CS Covid-19)”	Expert	1W; 7M (8 total) 12.5%W	Yes, Pr Marielle Bouyou Akothe	Yes	No, Julien Nkoghe Bekale	N/A
41	Ghana^98 99^	**Inter-Ministerial Coordinating Committee (IMCC) on Decentralisation (IMCCoD**)	Decision-making	3W; 7M (10 total) 30%W	Unknown	Yes	No, Nana Akufo-Addo	N/A
42	Greece^100^	**Commission for the Management of Emergency Events due to Infectious Diseases**	Decision-making and expert	8W; 18M (26 total) 30.8%W	Unknown	Yes	No, Kyriakos Mitsotakis	N/A
43	Grenada^101–103^	**Name unknown**	Decision-making and expert	0W; 5M (five total) 0%W	No, unknown	Yes	No, Keith Mitchell	N/A
44	Guinea^104–106^	**Scientific Council for Response to the Coronavirus Disease Pandemic** “Conseil scientifique de riposte contre la pandémie de la maladie à coronavirus (COVID-19)”	Expert	3W; 14M (17 total) 17.6%W	Yes, Pr Yolande Izazy	Yes	No, Ibrahima Kassory Fofana	N/A
		**Interministerial Committee for the Fight against the Coronavirus-19 epidemic** “Comité Interministerial de Lutte contre L'épidémie de Coronavirus-19”	Decision-making	3W; 19M (22 total) 13.6% W	No, Ibrahima Kassory Fofana	No		N/A
45	Haiti^107^	**Scientific unit to fight against the coronavirus** “Cellule scientifique pour lutter contre le coronavirus”	Expert	2W; 12M (14 total) 14.3%W	No, Patrick Dely	Yes	No, Joseph Jouthe	N/A
		**Communication unit on the pandemic** “Cellule de communication sur la pandémie”	Other	1W; 10M (11 total) 9.1%W	No, Eddy Jackson Alexis	Yes	N/A
46	Hungary 86 87	**Operational Staff (Coronaviral Defence Operational Staff**) “Koronavírus-fertőzés Elleni Védekezésért Felelős Operatív Törzs”	Decision-making	1W; 14M (15 total) 6.7%W	No, Sándor Pintér and Miklós Kásler	Yes	No, Viktor Orbán	N/A
47	India^108^	**COVID-19 Task Force**	Decision-making and expert	2W; 14M (16 total) 12.5%W	No, Narendra Modi	Yes	No, Narendra Modi	N/A
48	Iraq^109 110^	**High Committee for the National Health and Safety to combat Coronavirus** اللجنة العليا للصحة والسلامة الوطنية	Decision-making	0W; 24M (24 total) 0%W	No, Adel Abdul Mahdi	Yes	No, Mustafa Al-Kadhimi	N/A
49	Ireland^111–113^	**National Public Health Emergency Team (NPHET**)	Decision-making	13W; 19M (32 total) 40.6%W	No, Tony Holohan	Yes	No, Micheál Martin	N/A
		**Expert advisory group on COVID-19**	Expert	8W; 10M (18 total) 44.4%W	No, Cillian de Gascun	Yes	No	N/A
50	Italy^114–117^	**Operational Committee on Coronavirus for Civil Protection** “Comitato tecnico Scientifico per l'emergenza Coronavirus”	Decision-making	2W; 5M (7 total) 28.6%W	No, Giuseppe Conte	Yes	No, Giuseppe Conte	N/A
		**Scientific Technical Committee** “Comitato Tecnico Scientifico”	Expert	0W; 7M (7 total) 0%W	No, Agostino Miozzo	Yes	N/A
		**Task force tech anti-Covid-19**	Other	18W; 56M (74 total) 24.3%W	Yes, Fidelia Cascini (co-chair)	Yes	N/A
51	Jamaica^118^	**COVID-19 Economic Recovery Task Force**	Decision-making	4W; 18M (22 total) 18.18%W	No, Nigel Clarke	Yes	No, Andrew Holness	N/A
52	Japan^119 120^	**Novel Coronavirus Infectious Disease Control Expert Committee**	Expert	2W; 10M (12 total) 16.7%W	Unknown	Yes	No, Shinzo Abe	N/A
		**Special mission task force on remote medicine**	Other	4W; 4M (8 total) 50%W	Unknown	Yes	N/A
53	Kenya^121 122^	**National Emergency Response Committee**	Decision-making	4W; 17M (21 total) 19%W	No, Mutahi Kagwe	Yes	No, Uhuru Kenyatta	N/A
54	Lao People’s Democratic Republic^123^	**National Taskforce Committee for Covid-19 Prevention and Control**	Decision-making	0W; 11M (11 total) 0%W	No, Somdy Douangdy	Yes	No, Thongloun Sisoulith	N/A
55	Libya^124^	**Supreme Committee for Combating COVID-19** اللجنة العليا لمكافحة وباء «كورونا	Decision-making	1W; 3M (4 total) 25%W	No, Abdel Razek al-Nadhuri	Yes	No, Fayez al-Sarraj	N/A
		**Medical Advisory Committee** اللجنة الطبية الاستشارية	Expert	2W; 9M (11 total) 18.18%W	Yes, Fathia Al-Uraibi and Ahmed Al-Hassi	Yes		N/A
56	Lithuania^125 126^	**Committee responsible for COVID-19 management** (Official name unclear)	Decision-making	0W; 11M (11 total) 0% W	No, Saulius Skvernelis	Yes	No, Saulius Skvernelis	N/A
57	Luxembourg^127^	**Advisory Council to accompany the measures decided as part of the fight against COVID-19**	Expert	3W; 5M (8 total) 37.5%W	Unknown	Yes	No, Xavier Bettel	N/A
58	Malawi^128^	**Special Cabinet Committee on Coronavirus**	Decision-making	1W; 10M (11 total) 9.1%W	No, Jappie Mtuwa Mhango	Yes	No, Lazarus McCarthy Chakwera	N/A
59	Mali^129 130^	**Crisis Committee** “Le Comité de crise”	Decision-making	0W; 12M (12 total) 0%W	No, Akory Agiknane	No	No, Boubou Cissé	N/A
		**Scientific and Technical Committee of the National Public Health Institute** “Comité Scientifique et Technique de l’Institut National de Santé Publique –INSP”	Expert	1W; 9M (10 total) 10%W	No, Ousmane Koita	No		N/A
60	Myanmar^131 132^	**Coronavirus Disease 2019 (COVID-19) Control and Emergency Response Committee**	Decision-making	0W; 10M (10 total) 0%W	No, U Myint Swe	Yes	Yes, Aung San Suu Kyi	N/A
61	Netherlands^133^	**Outbreak Management Team** (No Dutch name)	Expert	6W; 3M (9 total) 67%	No, Jaap van Dissel	Yes	No, Mark Rutte	The list here consists of the permanent members and excludes the invited members.
62	New Zealand^134^	**Epidemic Response Select Committee**	Expert	4W; 7M (11 total) 36.4%W	Unknown	Yes	Yes, Jacinda Ardern	The committee was disestablished on 26 May 2020.
63	Niger^135^	**The Advisory Committee** “Le Comité Consultatif”	Expert	1W; 12M (13 total) 7.7%W	No, Alkache Alhada	No	No, Brigi Rafini	N/A
64	Nigeria^136^	**Presidential Task Force for the Control of the Coronavirus**	Decision-making	1W; 11M (12 total) 8.3%W	No, Garbu Shehu	Yes	No, Muhammadu Buhari	N/A
65	Oman^137^	**High level Ministerial Committee on Corona Development** اللجنة العليا المكلفة ببحث آلية التعامل مع التطورات الناتجةعن انتشار فيروس كورونا كوفيد19	Decision-making	1W; 9M (10 total) 10%W	No, Hammoud bin Faisal Al Busaidi	No	No, Haitham bin Tariq	N/A
66	Paraguay^138^	**Emergency Operations Centre of the Ministry of Public Health and Social Welfare to give a national response to the eventual Coronavirus pandemic** “Centro de Operaciones de Emergencia del Ministerio de Salud Pública y Bienestar Social para dar respuesta nacional de la eventual Pandemia por Coronavirus”	Decision-making and expert	2W; 6M (8 total) 25%W	Unknown	Yes	No, Mario Abdo Benítez	N/A
67	Philippines^139^	**Inter-Agency task force**	Decision-making	0W; 4M (4 total) 0%W	No, Francisco T. Duque, Karlo Nograles, and Roy Cimatu	No	No, Rodrigo Duterte	N/A
		**National task force Covid-19** “National Disaster Risk Reduction and Management Council - NDRRMC)”	Decision-making	0W; 4M (4 total) 0%W	No, Delfin Negrillo Lorenzana	No		N/A
68	Portugal^140 141^	**Task Force for operationalisation and implementation of measures for prevention and control of infection with new Coronavirus – COVID-19** “Task Force para a operacionalização e a implementação de medidas para prevenção e controlo da infeção por novo Coronavírus - COVID-19”	Decision-making & expert	44W; 32M (76 total) 57.9% W	Yes, Graça Freitas	Yes	No, António Costa	N/A
		**National Council for Public Health** “Conselho Nacional de Saúde”	Decision-making and expert	6W; 15M (21 total) 28.6%W	Unknown	Yes	N/A
69	Qatar^142^	**Supreme Committee on Disaster Management** “للجنة العليا لإدارة الأزماتا”	Decision-making	1W; 15M (16 total) 6.25%W	No, Sheikh Khalid bin Khalifa bin Abdul Aziz Al Thani	Yes	No, Sheikh Khalid bin Khalifa bin Abdul Aziz Al Thani	N/A
70	Saudi Arabia^143^	**Designated Committee to Monitor Corona Pandemic** اللجنة المعنية بمتابعة مستجدات الوضع الصحي لفيروس كورونا	Decision-making	0W; 17M (17 total) 0.0%W	No, Unknown	Yes	No, Salman bin Abdulaziz Al Saud	N/A
71	Serbia^144^	**Crisis Team for the Control of Infectious Diseases COVID-19** “Кризни штаб за сузбијање заразне болести COVID-19”	Decision-making	6W; 21M (27 total) 16.7%W	Yes, Ana Brnabić, and Zlatibor Lonĉar (co-chairs with two others)	Yes	Yes, Ana Brnabić	This list excludes the additional engaged experts, only including the formal members.
72	Singapore^145^	**Multi-Ministry Taskforce on Wuhan Coronavirus**	Decision-making	1W; 10M (11 total) 9.1%W	No, Gan Kim Yong and Lawrence Wong	Yes	No, Lee Hsien Loong	N/A
73	South Africa^146 147^	**Ministerial Advisory Committees on COVID-19**	Expert	30W; 24M (54 total) 55.6%W	No, Salim S. Abdool Karim	Yes	No, Cyril Ramaphosa	N/A
74	South Sudan^148^	**High Level Task Force Committee to take Extra Precautionary Measures in Combating the Spread of Coronavirus Disease (COVID-19**)	Decision-making	3W; 13M (16 total) 18.8%W	No, Salva Kiir Mayardit	No	No, Salva Kiir Mayardit	N/A
75	Spain^149 150^	**Scientific Technical Committee COVID-19** *“el Comité Científico Técnico COVID-19*“	Expert	3W; 4M (7 total) 42.9%W	Unknown	Yes	No, Pedro Sánchez	N/A
76	Sri Lanka^151 152^	**Presidential Task Force on economic revival and poverty eradication**	Other	1W; 30M (31 total) 3.2%W	No, Basil Rajapaksa	Yes	No, Gotabaya Rajapaksa	N/A
77	Sweden^153^	**Management Team of the Public Health Agency** “Folkhälsomyndighetens ledningsgrupp”	Unclear	5W; 2M (7 total) 71.4%W	No, Johan Carlson	Yes	No, Stefan Löfven	N/A
78	Switzerland^154–156^	**Swiss National COVID-19 Science Task Force**	Expert	2W; 5M (7 total) 28.6%W	No, Matthias Egger	Yes	Yes, Simonetta Sommaruga	N/A
		**Corona Crisis Team of the Federal Council** “Krisenstab des Bundesrats Corona”	Decision-making	2W; 12M (14 total) 14.3%W	Yes, Simonetta Sommaruga	Yes		N/A
79	Thailand^157^	**National committee for controlling the spread of COVID-19** “คณะกรรมการแห่งชาติเพื่อควบคุมการแพร่กระจายของ COVID-19”	Decision-making	0W; 28M (28 total) 0%W	No, Prayut Chan-o-cha	No	No, Prayut Chan-o-cha	N/A
80	Togo^158 159^	**COVID-19 Pandemic Crisis Management Unit** “Cellule sectorielle de la gestion de la crise à la Pandémie de covid-19”	Decision-making and Expert	2W; 9M (11 total) 18.2%W	Unknown	Yes	No, Komi Sélom Klassou	N/A
81	Trinidad & Tobago^160^	**Team for COVID-19 ‘Road to Recovery’** (Official name unknown)	Decision-making	1W; 21M (22 total) 4.5%W	No, Keith Rowley	Yes	No, Keith Rowley	N/A
82	Turkey^161^	**Coronavirus Scientific Committee** “Koronavirüs Bilim Kurulu”	Expert	14W; 22M (36 total) 39.9%W	Unknown	Yes	No, Recep Tayyip Erdoğan	N/A
83	Uganda^162^	**National Response Fund to COVID-19**	Other	3W; 12M (15 total) 20%W	No, Emmanuel Katongole	Yes	No, Ruhakana Rugunda	Information was obtained through Wikipedia and sources references on the Wikipedia page.
84	United Kingdom^163–165^	**New and Emerging Respiratory Virus Threats Advisory Group**	Expert	2W; 14M (16 total) 12.5%W	No, Peter Horby	Yes	No, Boris Johnson	N/A
		**Advisory Committee on Dangerous Pathogens**	Expert	3W; 13M (16 total) 18.8%W	No, Thomas Evans	Yes		N/A
		**Joint Committee on Vaccination and Immunisation**	Expert	4W; 16M (20 total) 20%W	No, Andrew Pollard	Yes		N/A
85	United States^166–168^	**White House Coronavirus Task Force**	Decision-making	2W; 20M (22 total) 9.1%W	No, Donald Trump	Yes	No, Donald Trump	N/A
		**Centres for Disease Control and Prevention (CDC) COVID-19 Response Team**	Expert	14W; 3M (17 total) 82.4%W	Unknown	Yes		N/A
86	Uruguay^169^	**Committee of Scientific Experts in Crisis Management** “Comité de Expertos Científicos en Gestión de la Crisis”	Expert	1W; 6M (7 total) 14.3%W	No, Julio Rolon Vicioso	Yes	No, Luis Lacalle Pou	N/A
87	Vietnam^170^	**National Steering Committee for COVID-19 Prevention and Control** “Ban chỉ đạo quốc gia về phòng chống và kiểm soát COVID-19”	Decision-making	1W; 13M (14 total) 7.1%W	No, Đỗ Xuân Tuyên	No	No, Nguyễn Xuân Phúc	N/A

Men were overrepresented in global task forces to a similar extent to that of national task forces (table 2). For instance, the WHO’s first, second and third International Health Regulations Emergency committees consisted of 23.8%, 23.8% and 37.5% women, respectively. Expert groups, compared with decision-making committees, more frequently had higher proportions of women or gender parity, reflecting potential societal biases and stereotypes in terms of gender roles. In the USA, for example, the White House Coronavirus Task Force consists of 9.1% women, whereas the chief public health agency’s COVID-19 Response Team contains 82.4% women. Evidently, COVID-19 governance followed the usual modus operandi, despite numerous global and national commitments to gender-responsive health governance.

**Table 2:** Identified global COVID-19 task forces

#	Name of the task force convened	Gender	Women head of force	Public	Women head of international body	Note
1	World Health Organization (WHO) – China Joint Mission Team 171	3W; 22 M (25 total) 12% W	No, Bruce Aylward	Yes	No, Tedros Adhanom Ghebreyesus	List includes members and advisors
2	WHO International Health Regulations (IHR) Emergency Committee for Pneumonia due to the Novel Coronavirus 2019-nCoV 172	5W; 16 M (21 total) 23.8% W	No, Didier Houssin	Yes	No, Tedros Adhanom Ghebreyesus	List includes members and advisors
3	WHO International Health Regulations Second Emergency Committee 173	5W; 16M (21 total) 23.8%W	No, Didier Houssin	Yes	No, Tedros Adhanom Ghebreyesus	List includes members and advisors
4	WHO International Health Regulations Third Emergency Committee for COVID-19 174	12W; 20 M (32 total) 37.5% W	No, Didier Houssin	Yes	No, Tedros Adhanom Ghebreyesus	List includes members and advisors
5	European Union (EU) COVID-19 Coordinating Response Team 175	4W; 2M (6 total) 66.7% W	Yes, Ursula von der Leyden	Yes	Yes, Ursula von der Leyden	N/A
6	EU Commission’s advisory panel on COVID-19 176	2W; 6M (8 total) 25% W	Unknown	Yes	Yes, Ursula von der Leyden	N/A
7	Africa Taskforce on Coronavirus Preparedness and Response 177	2W; 14M (16 total) 12.5% W	No, John Nkengasong	Yes	N/A	Joint effort of the African Union and Africa CDC

This analysis was based on a large-scale effort collecting data on COVID-19 global and national decision-making and expert bodies for 193 UN Member States through a crowdsourcing effort, targeted grey literature searches, and outreach to national governments or World Health Organization (WHO) country offices. Data collection was completed June 2020. Gender was determined based on prefixes, pronouns and online bibliographies (table 3). Most information pertaining to task force construction, leadership and membership criteria (eg, expertise) was not easily accessible nor publicly available, impeding research and, ultimately, the ability to hold countries accountable to previously made commitments.

**Table 3:** Identification of national COVID-19 task forces

Category	#	UN member states
Able to identify complete task force information of at least one task force formed in response to COVID-19.	87	Albania; Algeria; Argentina; Armenia; Australia; Austria; Bahamas; Bahrain; Bangladesh; Belgium; Benin; Bhutan; Bolivia; Botswana; Brazil; Bulgaria; Burkina Faso; Cape Verde; Canada; Chad; Chile; China; Colombia; Comoros; Congo; Costa Rica; Côte d'Ivoire; Cuba; Cyprus; Democratic People’s Republic of Korea; Democratic Republic of the Congo; Djibouti; Dominican Republic; Ecuador; Estonia; Eswatini; Ethiopia; Finland; France; Gabon; Ghana; Greece; Grenada; Guinea; Haiti; Hungary; India; Iraq; Ireland; Italy; Jamaica; Japan; Kenya; Lao People’s Democratic Republic; Libya; Lithuania; Luxembourg; Malawi; Mali; Myanmar; Netherlands; New Zealand; Niger; Nigeria; Oman; Paraguay; Philippines; Portugal; Qatar; Saudi Arabia; Serbia; Singapore; South Africa; South Sudan; Spain; Sri Lanka; Sweden; Switzerland; Thailand; Togo; Trinidad & Tobago; Turkey; Uganda; United Kingdom; United States; Uruguay; Vietnam
Able to identify the name of at least one task force formed in response to COVID-19, but not the task force composition.	44	Afghanistan; Angola; Antigua and Barbuda; Azerbaijan; Belize; Burundi; Cambodia; Central African Republic; Equatorial Guinea; Fiji; Gambia; Guinea-Bissau; Iceland; Indonesia; Jordan; Latvia; Lebanon; Liberia; Liechtenstein; Madagascar; Maldives; Malaysia; Mauritius; Micronesia; Mongolia; Morocco; Mozambique; Namibia; Nauru; Nepal; Pakistan; Republic of Korea; Republic of Moldova; Rwanda; Saint Kitts and Nevis; Saint Lucia; Saint Vincent and the Grenadines; Samoa; Senegal; Sierra Leone; Suriname; Tonga; Tunisia; Zimbabwe
Able to identify the existence of at least one task force formed in response to COVID-19 but not the name or the task force composition.	7	Denmark; Kiribati; Kuwait; Mexico; Seychelles; Solomon Islands; Somalia
Not able to identify the existence of at least one task force formed in response to COVID-19.	55	Andorra; Barbados; Belarus; Bosnia and Herzegovina; Brunei Darussalam; Cameroon; Croatia; Czech Republic; Dominica; Egypt; El Salvador; Eritrea; Georgia; Germany; Guatemala; Guyana; Honduras; Iran; Israel; Kazakhstan; Kyrgyzstan; Lesotho; Malta; Marshall Islands; Mauritania; Monaco; Montenegro; Nicaragua; North Macedonia; Norway; Palau; Papua New Guinea; Panama; Peru; Poland; Romania; Russian Federation; San Marino; Sao Tome and Principe; Slovakia; Slovenia; Sudan; Syrian Arab Republic; Tajikistan; Timor-Leste; Turkmenistan; Tuvalu; Ukraine; United Arab Emirates; United Republic of Tanzania; Uzbekistan; Vanuatu; Venezuela; Yemen; Zambia

## The default governance mode is losing out on key perspectives and expertise

While current evidence suggests direct COVID-19 severity and mortality is higher for men, women are disproportionately burdened by compounded social and economic impacts.^1 2^ Decision-making bodies which are neither inclusive nor diverse can easily overlook the reality that COVID-19 acts as a multiplier of pre-existing gender-based inequities. Many governments established COVID-19 response measures which disregarded women’s higher levels of income loss, expanded and unpaid family care responsibilities, and gendered poverty rates. Ignorance of these implications exacerbates (lifetime) poverty and hunger.^3^ Response measures often do not account for women’s increased exposure to domestic and sexual violence or their loss of access to essential health services. Furthermore, many lockdown policies do not consider maternal and reproductive health service as essential care.^4–6^ Experiences from Ebola and Zika demonstrated rises in maternal morbidity and mortality, unwanted pregnancies and unsafe abortions.^3^ Despite being publicly praised with hollow applause, the majority of COVID-19 frontline health and social workforce are women who are underpaid, unpaid or are not recognised as essential at all. Failure to adequately provide resources and personal protective equipment exacerbates disease transmission and disproportionately harms workers in the health and social care sectors, which are predominated by women.^7^ The situation is even more dire for marginalised individuals, such as those identifying as non-binary, transgender or genderqueer, as they are forced to navigate the discriminatory impacts of gender-based quarantine guidelines, which authorise specific days when women or men are allowed in public. As seen in Panama, this often led to harassment, abuse, arrest and fines of transgender people who were wrongfully profiled.^8–10^

## Effective change calls for bold solutions

The exclusion of women and gender minorities stems from a host of factors including inherent conscious and unconscious biases, discrimination, workplace culture and gendered expectations. Unfortunately, this is not new. Although women comprise 70% of the global health workforce, they hold only 25% of senior decision-making roles. Women from the Global South are particularly underrepresented at global level holding less than 5% of senior leadership roles. This exclusion creates a vicious cycle where perspectives and knowledge of large segments of the population continue to be excluded.^11 12^ One cannot expect a different result by replicating this same broken cycle over and over again. A ‘new default’ mode of diverse and intersectional governance is sorely needed to face future crises head-on and guide a healthy and equitable COVID-19 recovery. Reaching a critical mass of women in leadership – even as result of intentional selection or quotas – benefits governance processes through the disruption of groupthink, the introduction of novel viewpoints, a higher quality of monitoring and management, more effective risk management and robust deliberation.^13^

Interestingly, countries with women leaders have been associated with implementing particularly effective COVID-19 responses and have been better at reducing COVID-19 negative impacts (fewer deaths per capita, a lower peak in daily deaths and lower excess mortality). A recent study indicated that countries with women in positions of leadership suffered six times fewer deaths from COVID-19 as countries with governments led by men.^14^ Recognising the effectiveness of countries led by women may help in understanding the underlying prerequisites of effective leadership. Societies who elect female leaders may share a different set of values and perspectives, including gender equality, than more traditional societies.^15^ Countries where women lead seem to have political institutions and cultures that have prepared for inclusive governance being practised prior to COVID-19, influencing their COVID-19 response.

Gender quotas can establish a standard to redress inequalities in the public realm and enable more effective decision-making through gender parity. Increasing women’s representation is a key step towards addressing inequalities- but it cannot stop there.^16 17^ More women in leadership positions does not necessarily lead to changes in social norms nor does it guarantee the gender-responsive, gender-mainstreamed policies needed to mitigate the gendered vulnerabilities of pandemics. Women are not automatically gender-inclusive advocates, nor are men inevitably gender-exclusive.^17 18^ Furthermore, gender intersects with additional factors that act as significant barriers to healthcare access and participation. This requires recognising inequities across ability, race, income, ethnicity, class, religion and geography, and intentionally prioritising programmes and resources with an intersectional, inclusive lens. It is critical to highlight the gender-specific impacts of health threats, collect gender disaggregated data (as done for COVID-19 by Global Health 50/50)^19^ and leverage female experts (like WGH Operation 50/50).^20^ Claiming to not find any qualified women in global health is ultimately an unjustifiably poor excuse for excluding diverse perspectives. Systemic and cultural change must address traditional norms and attitudes, and embrace holistic gender-mainstreaming practices. This deep-rooted change is critical to ensure that health services and policies mitigate the adverse socio-economic impacts of COVID-19 and adequately meet the needs and safety of all populations.^17 21^

## Going further than gender binaries

Despite employing colloquial binary terms such as ‘men’ and ‘women’ to denote gender, we reiterate that gender is non-binary, socially produced, self-identified and complex. In a non-pandemic scenario, we would have sought to conduct a survey to self-identify gender, with appropriate ethics review, privacy and data protections in place. By relying on binary definitions of “gender,” research initiatives (such as this one) and governance, emphasise the inability of current data to produce results that include the full gender spectrum. This means an entire segment of the population is misrepresented and side-lined from policy decisions that affect them. Promoting and integrating mechanisms that ensure inclusive intersectional data collection is one of the systemic changes needed for fair governance.

## Inclusivity and transparency should be at the core of the 'new normal’

Our data exhibit what has become a disturbingly accepted pattern in global health governance. Collective efforts in policy-making continue to overlook opportunities to create inclusive and comprehensive decision-making, echoing gender inequalities in other areas such as academia and the sciences.^22^ The COVID-19 pandemic response requires inclusion of diverse perspectives, experiences and expertise in global health leadership. First, international and national task forces need to ensure diversity, particularly across gender, but also in terms of ethnic, racial, cultural, geographic and disability groups in decision-making and expert advisory bodies. Increasing representation and gender parity is a first step, but functional health systems require radical and systemic change that ensures gender-inclusive and intersectional practices are the norm – rather than the exception. Second, quick action in emergency scenarios is repeatedly used as a justification to sidestep transparency and restrict communication in the name of health security. Crises are precisely when transparent procedures and clear communication are required the most. Rather than relying on closed-door governance, open and transparent communication and decision-making should become the norm. Third, data collection and governance policies need to go beyond binary representation in order to produce results that are inclusive of the full gender spectrum.

A future with resilient health systems depends on radical action to establish decision-making groups that reflect the populations they represent, in the time of COVID-19 and beyond. Leaving these voices unheard today sets a precedent for continued silence in the years to come.
